# Supplementary material for: Integration of Transcriptome and Metabolome Reveals Wax Serves a Key Role in Preventing Leaf Water Loss in Goji (Lycium barbarum)
Source: Int J Mol Sci. 2024 Oct 11;25(20):10939. doi: 10.3390/ijms252010939 (PMC11507121; doi:10.3390/ijms252010939)
Supplement: Supplementary file 1 [file ijms-25-10939-s001.zip › ijms-3162504-supplementary.pdf]

Table S1. Primers used in this study

Purpose	Primer	Primer Sequence (5'-3')
Quantitative real-time PCR	*qLbaCER1.1-F*	GCAATGCTAGTTCCTCCTTCTCTG
	qLbaCER1.1-R	ACATGCACAATTCCAGCCACTC
	qLbaCER1.2-F	CCTCTAAACCTGGCATTCTCACTG
	qLbaCER1.2-R	AATGTCCCTCTGGCTTTCATCTTTG
	qLbaMYBS3-F	TCAAGGACTCCCACCCAAGTAGC
	qLbaMYBS3-R	AGTAGCCGTGTCAGCAACAATGTC
	qLbaMYB3-F	GAAGGGGACTTGGACTCCTGAGG
	qLbaMYB3-R	GCTCTTTCCACACCTTGCTAGACC
	qLbaMYB48-F	ACGCCTTGTTCTTCAACTCCACTC
	qLbaMYB48-R	ATCTTGGGCCTGCTTTCTCATGTG
	qLbaMYB60-F	TGGGTTGATGAGGTGCAGCAAAAG
	qLbaMYB60-R	AGCTATGGCTGCCCATTTGTTACC
	qLbaMYB306-F	AATCGTCGTCTCCATGCCAATCG
	qLbaMYB306-R	ACAGCGAGTCCAAACCTTTTCCAG
	qLbaWRKY68-F	ACGCCATGTACCCCAAACTGTTC
	qLbaWRKY68-R	GCTCTCTCTGCTGCTTCTTCTGAC
Reference Genes	*LbaRh37-F*	GCAGGCAAGTCAGGATTAGCA
	LbaRh37-R	CGCATAACGAGTCAACCATTCAG

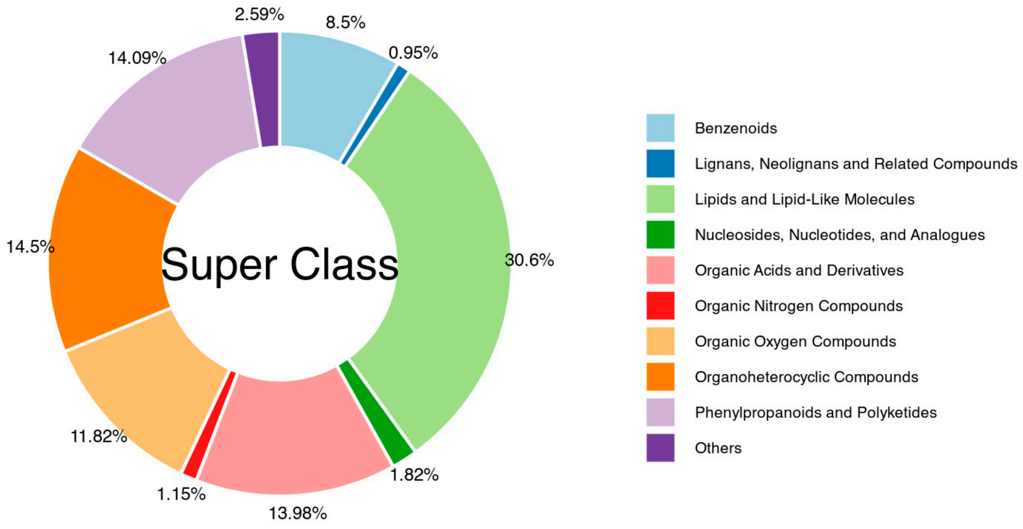

Figure S1. Classification of Metabolites Diagram. A total of 5552 metabolites were detected in the positive and negative ion modes, which can be divided into 10 categories.

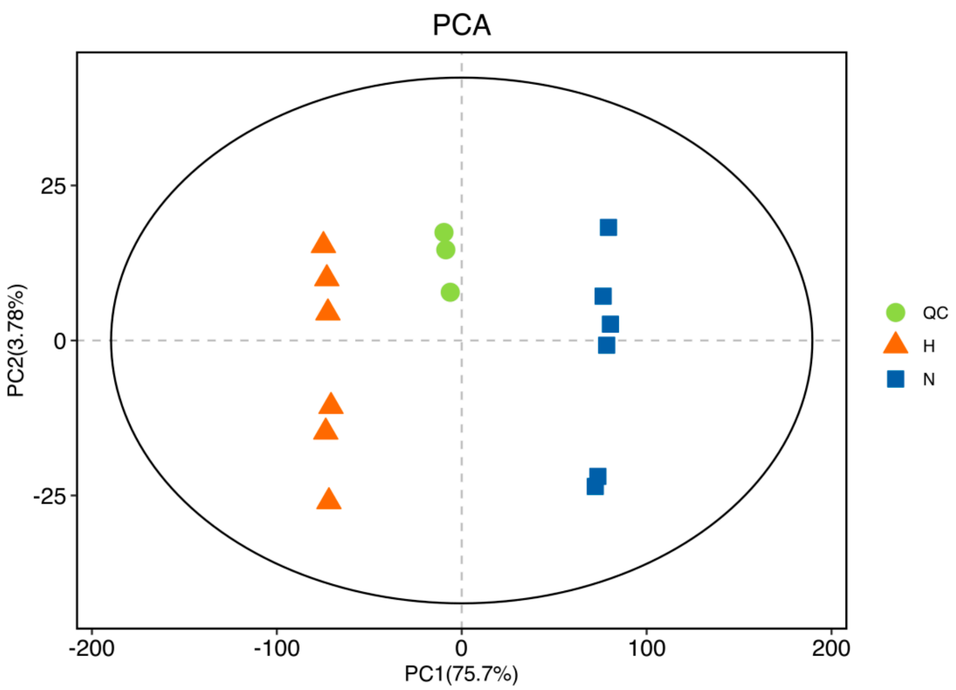

Figure S2. PCA Principal Component Analysis plot.The horizontal axis PC1 represents the explained variance of the first principal component, and the vertical axis PC2 represents the explained variance of the second principal component. Each point in the figure represents a sample.The high clustering of the two types of goji leaves' metabolites indicates significant differences in the metabolic profiles among the samples. ' N ' represents ‘Ningqi I’ goji ; 'H ' represents ‘Huangguo’ goji, ‘QC’ represents Quality Control.

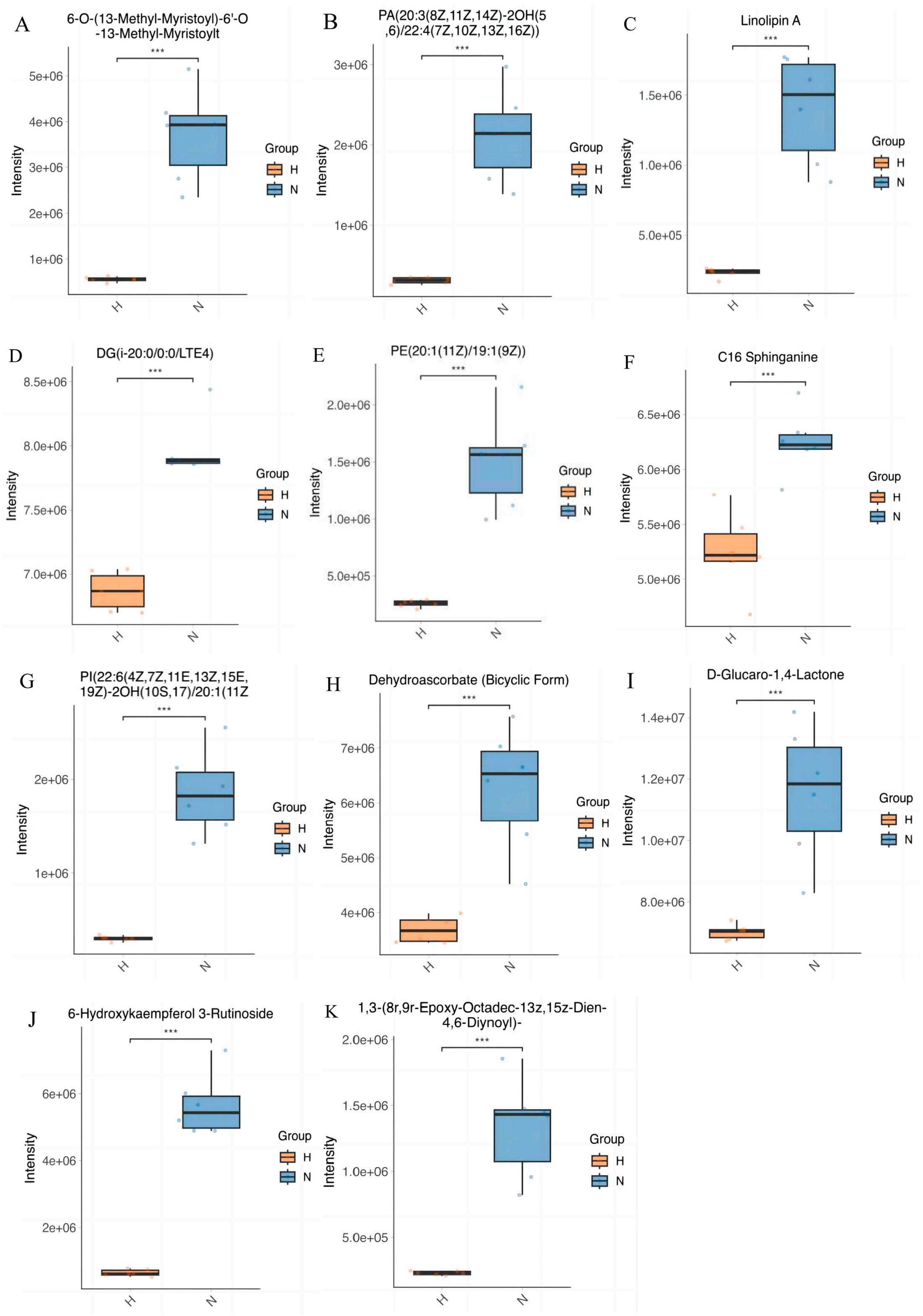

Figure S3. The box plot of the content variation of 11 metabolites in ‘Ningqi I’ goji and ‘Huangguo’ goji. The content of 11 metabolites is significantly higher in ‘Ningqi I’ goji than in ‘Huangguo’ goji. ' N ' represent ‘Ningqi I’ goji ; 'H ' represent ‘Huangguo’ goji.
